# A network pharmacology-based study on the anti-hepatoma effect of Radix *Salviae Miltiorrhizae*

**DOI:** 10.1186/s13020-019-0249-6

**Published:** 2019-08-06

**Authors:** Yi Luo, Yu Feng, Lei Song, Gan-Qing He, Sha Li, Sha-Sha Bai, Yu-Jie Huang, Si-Ying Li, Mohammed M. Almutairi, Hong-Lian Shi, Qi Wang, Ming Hong

**Affiliations:** 1 Institute of Clinical Pharmacology, Guangzhou University of Chinese Medicine, Guangzhou, 510405 China; 2 Department of Traumatology, General Hospital of Ningxia Medical University, Yinchuan, 750004 China; 3 Department of Orthopaedics and Traumatology, Li Ka Shing Faculty of Medicine, The University of Hong Kong, Hong Kong, China; 4 Department of Gastroenterology, Second Affiliated Hospital of Guangzhou Medical University, Guangzhou, 501260 China; 5 School of Chinese Medicine, Li Ka Shing Faculty of Medicine, The University of Hong Kong, Hong Kong, China; 6 Department of Pharmacology & Toxicology, University of Kansas, Lawrence, KS USA

**Keywords:** Radix *Salviae Miltiorrhizae*, Hepatocellular carcinoma, Network pharmacology

## Abstract

**Background:**

Radix *Salviae Miltiorrhizae* (RSM), a well-known traditional Chinese medicine, has been shown to inhibit tumorigenesis in various human cancers. However, the anticancer effects of RSM on human hepatocellular carcinoma (HCC) and the underlying mechanisms of action remain to be fully elucidated.

**Methods:**

In this study, we aimed to elucidate the underlying molecular mechanisms of RSM in the treatment of HCC using a network pharmacology approach. In vivo and in vitro experiments were also performed to validate the therapeutic effects of RSM on HCC.

**Results:**

In total, 62 active compounds from RSM and 72 HCC-related targets were identified through network pharmacological analysis. RSM was found to play a critical role in HCC via multiple targets and pathways, especially the EGFR and PI3K/AKT signaling pathways. In addition, RSM was found to suppress HCC cell proliferation, and impair cancer cell migration and invasion in vitro. Flow cytometry analysis revealed that RSM induced cell cycle G2/M arrest and apoptosis, and western blot analysis showed that RSM up-regulated the expression of BAX and down-regulated the expression of Bcl-2 in MHCC97-H and HepG2 cells. Furthermore, RSM administration down-regulated the expression of EGFR, PI3K, and p-AKT proteins, whereas the total AKT level was not altered. Finally, the results of our in vivo experiments confirmed the therapeutic effects of RSM on HCC in nude mice.

**Conclusions:**

We provide an integrative network pharmacology approach, in combination with in vitro and in vivo experiments, to illustrate the underlying therapeutic mechanisms of RSM action on HCC.

**Electronic supplementary material:**

The online version of this article (10.1186/s13020-019-0249-6) contains supplementary material, which is available to authorized users.

## Background

Hepatocellular carcinoma (HCC) is the most frequent primary liver malignancy and is a major health problem worldwide [1]. In 2017, 782,000 cases were diagnosed and there were 746,000 deaths, and the incidence of HCC in worldwide continued to increase, with an age-adjusted incidence rising 10 per 100,000 individuals [2]. Chronic liver disease and cirrhosis remain the most significant risk factors for the development of HCC, of which hepatitis virus infection and excessive alcohol intake are the leading risk factors worldwide [3–5]. As patients in the early stages of HCC are generally asymptomatic, it is mainly diagnosed at an advanced stage, when the condition is incurable. While current treatment regimens have increased survival time for patients, the utilization of sorafenib has only increased survival by a few months [6]. In addition, sorafenib is associated with severe side-effects, which have limited the clinical use of the drug. Hence, new medicines with good efficacy and tolerability are urgently demanded.

In recent years, researchers have focused on developing a Traditional Chinese Medicine (TCM) for the treatment of HCC. Radix *Salviae Miltiorrhizae* (RSM), also known as Danshen in Chinese, is a widely used Chinese medicine, which has been applied clinically for more than 1000 years. Research has shown that RSM possesses a variety of pharmacological effects, including anti-platelet aggregation, anti-hypertension, anti-inflammation, and improvement of cerebral ischemia reperfusion injury, as well as cardiovascular protection [7–10]. Evidence accumulated in the last decade has demonstrated that RSM also demonstrates a significant anti-cancer effect against breast cancer, clear cell ovarian carcinomas, promyelocytic leukemia, and HCC [11–13]. Bae et al. [14] showed that *S. miltiorrhiza* Bunge might present a significant anticancer effect by inhibiting prostate cancer cell proliferation and inducing prostate cancer cell apoptosis. Kim et al. [15] revealed that *S. miltiorrhiza* extracts inhibited TPA-induced MCF-7 cell invasion and Matrix metalloproteinase-2 (MMP-9) expression by blocking the transcriptional activation of AP-1. The main active ingredients in Radix *S. miltiorrhizae* can be classified into two parts: hydrosoluble salvianolic acids (salvianolic acid A, salvianolic acid B, danshensu, rosmarinic acid) and liposoluble tanshinones (tanshinone IIA [TIIA], cryptotanshinone [CT], dihydrotanshinone I [DH-TI], tanshinone I [TI]). Previous studies have shown that TIIA is an active component of RSM, which inhibits human gastric cancer cells by decreasing HER2 and VEGFR protein levels, inhibits the Ras/Raf/MEK/ERK signaling pathway, and induces caspase-3, PARP activation, and apoptosis [16]. TIIA also decreases human cancer cell invasion and metastasis in the nude mice model. In addition, salvianolic acid B induces autophagy and exerts antitumor effects in colorectal cancer cells via inhibition of the AKT/mTOR pathway [17]. RSM and its active components may serve as a promising anti-cancer therapy; however, its pharmacological mechanisms of action have not been fully elucidated. Therefore, the aim of our research was to explore the anti-HCC effects of RSM extracts and explore the underlying mechanisms.

**Fig. 1:**
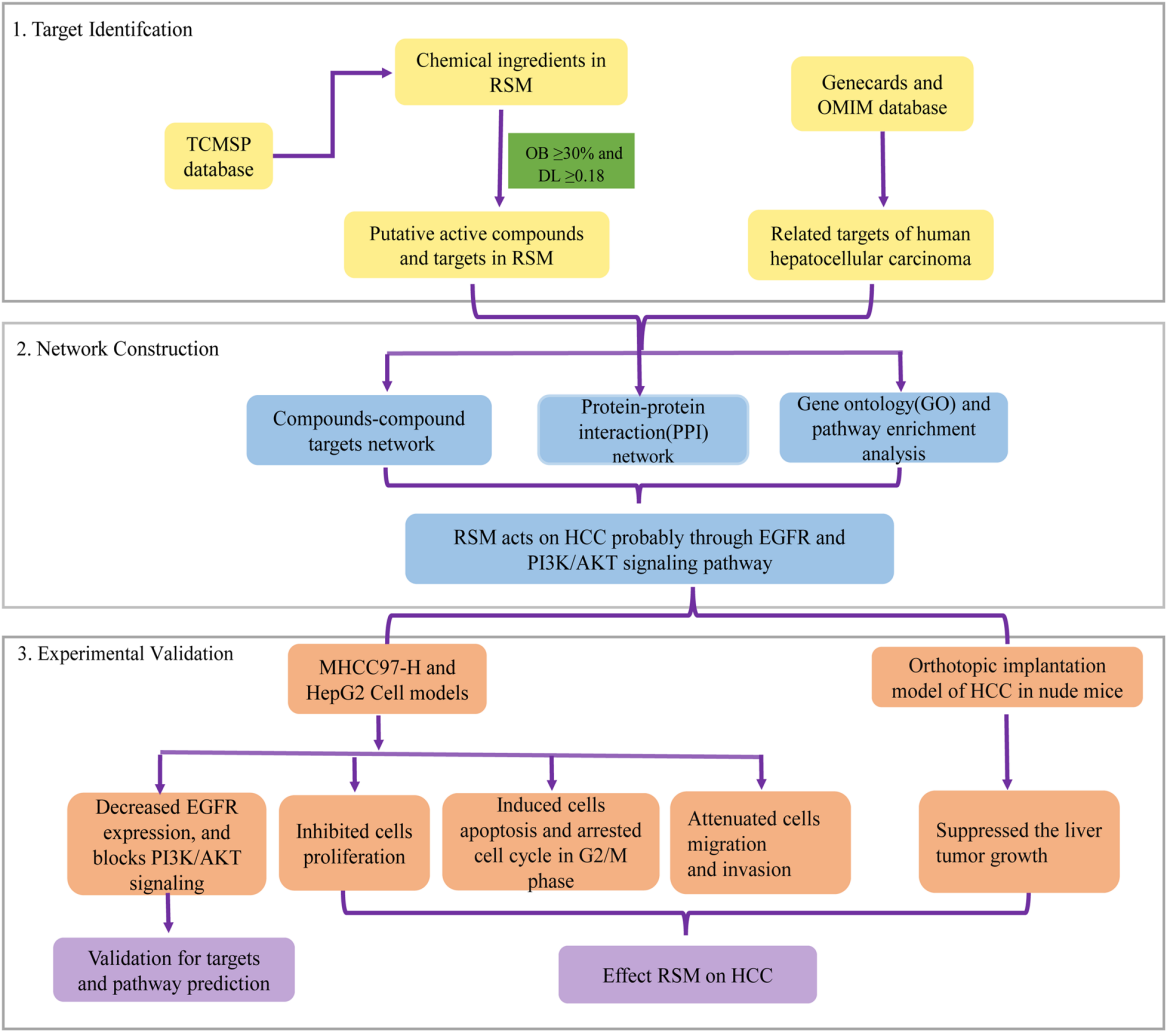
Flowchart showing the systems pharmacology approach for determining the pharmacological mechanisms of action of Radix *Salviae Miltiorrhizae* (RSM) on human hepatocellular carcinoma (HCC) by integrating target identification, network analysis, and experimental validation

TCM is typically associated with multiple targets, multiple pathways, and multiple mechanisms of actions, making it difficult to elucidate its effects. These greatly restrict the process of modernization and internationalization of Chinese medicine. The concept of network pharmacology derived from the introduction of systems biology and the application of bioinformatics, along with “omics” theory involved in modern genomic, proteomic, and metabolomic development. Network pharmacology was developed based on the rapid development of systems biology and computer technology. Based on the “disease-gene-target-drug” interaction network, network analysis was used to observe the intervention and influence of drugs on the disease network, and the synergistic effects of multiple drugs were determined [18, 19]. In recent years, network pharmacology has emerged as a powerful tool that can be integrated with pharmacology. It provides a novel tool and concept to evaluate the efficacy mechanisms of action of drugs. Combining the target database of TCM in various diseases with molecular verification provides evidence for the molecular target and its mechanism of action [20–23]. Thus, this study combines network pharmacology with experimental validation to clarify the potential mechanism of RSM against HCC and suggests a novel promising therapeutic strategy for the treatment of HCC (Fig. 1).

## Methods

The Minimum Standards of Reporting Checklist contains details of the experimental design, statistics, and resources used in this study.

### Compound database building

Traditional Chinese Medicine Systems Pharmacology (TCMSP) is a unique system pharmacology platform of Chinese herbal medicines, which captures the relationships between drugs, targets, and diseases. A catalog of chemical ingredients was generated from related articles and the natural products database of TCMSP (http://lsp.nwu.edu.cn/tcmsp.php) [24].

### Pharmacokinetic prediction

Absorption, distribution, metabolism, and excretion (ADME), an in silico evaluation model in pharmacokinetic research, was employed to select drugs. In this study, an in silico ADME-systems evaluation model including drug-likeness (DL) and oral bioavailability (OB) was applied to evaluate the potential bioactive components of RSM. OB is an essential parameter of orally administered drugs, and is used to assess the pharmacokinetics and drug-forming properties of drugs in vivo. The TCMSP database calculated OB values using OBioavail1.1 [25]. Drug-likeness (DL) is a qualitative concept used in drug design to evaluate how “drug-like” a molecule is with respect to factors such as bioavailability. DL is estimated from the molecular structure before the substance is synthesized and tested. To identify drug-like ingredients, the Tanimoto coefficient, *T* (*A*, *B*) = (*A* × *B*)/ (|*A*|2 + |*B*|2 − *A* × *B*) was applied to calculate the DL values of each ingredient in RSM by a database-dependent model. A presents the new compound, and B displays the average molecular properties of all compounds in the DrugBank database (http://www.drugbank.ca). In this study, molecules with DL ≥ 0.18, OB ≥ 30% were used for subsequent research, and others were excluded.

### Compound targets for RSM

A systematic drug targeting tool was used to predict the targets of candidate molecules, which efficiently combines genomic, chemical, and pharmacological information for drug targeting through Random Forest (RF) and Support Vector Machine (SVM) methods [26]. In this study, compound-target interactions with a RF score ≥ 0.7 and an SVM score ≥ 0.8 were regarded as potential targets for active molecules.

### Related targets of HCC

HCC-related genes were collected from two databases. 1. GeneCards (http://www.genecards.org) [27] is a searchable, integrative database of human genes that provides genomic, proteomic, transcriptomic, genetic, and functional information on all known and predicted human genes. The database automatically integrates gene-centric data from ~ 125 web sources, such as HGNC, NBCI, and Ensemble. 2. Online Mendelian Inheritance in Man (OMIM) (http://omim.org/) [28] is a comprehensive, authoritative compendium of human genes and genetic phenotypes, which is freely available and updated daily. We searched these databases with the keywords “hepatocellular carcinoma” and “hepatocarcinoma”.

### Construction of a protein–protein interaction (PPI) network

PPI data were derived from String (http://string-db.org/, ver.10) [29], an online database of known and predicted protein-protein interactions with the species limited to “Homo sapiens” and a confidence score > 0.4.

### Gene ontology (GO) and pathway enrichment analysis

The Database for Annotation, Visualization, and Integrated Discovery (DAVID; http://david.abcc.ncifcrf.gov/) [30, 31] was employed in GO and Kyoto Encyclopedia of Genes and Genomes (KEGG) pathway enrichment analysis.

### Network construction

The network was constructed as follows: (1) compound-compound target network of RSM; (2) PPI network. All visualized networks were constructed using software Cytoscape (http://cytoscape.org/, ver. 3.6.0), an open software platform for visualizing biological pathways, molecular interaction networks, and integrating these networks with annotations, gene expression profiles, and other state data.

### Reagents and antibodies

Fetal bovine serum (FBS), Dulbecco’s modified Eagle’s medium (DMEM), and penicillin–streptomycin were provided from Gibco. Cell culture supplies were obtained from Costar (Corning, USA). Antibodies, including anti-PI3-kinase p85-α (ab182651), anti-Bcl-2 (ab59348), HRP-conjugated goat anti-rabbit (ab6721), and anti-mouse IgG (ab6789) were from Abcam (Cambridge, UK). Antibodies, including anti-EGFR (#4267), anti-Bax (#2772), anti-AKT (#9272), anti-p-AKT (#4058), and β-actin (ab8227) were obtained from Cell Signaling Technology (Boston, USA).

### Preparation of ethanol extract from RSM

Dried roots of*. S. miltiorrhiza* Bunge were obtained from Xing Yuan Chun Pharmacy (Guangzhou, China). The original herb was identified by the Department of Traditional Chinese Medicine, Guangzhou University of Traditional Chinese Medicine, China. A voucher specimen was deposited at the public Herbarium of the Department of Traditional Chinese Medicine, Guangzhou University of Traditional Chinese Medicine. Samples (500 g) of dried roots of *S. miltiorrhiza* Bunge were extracted with 5 L of 95% ethanol using a refluxing method and then filtered. The filtrates were evaporated and lyophilized to obtain a dried powder. Dried powder obtained from the ethanol extracts of RSM was stored in 4 °C for future use.

### HPLC analysis

Ethanol extracts of Radix *S. miltiorrhizae* were qualitatively analyzed by high-performance liquid chromatography (HPLC). Shimadzu HPLC systems (LC-20AT, Japan), equipped with a CM-20A system controller, an infusion pump (LC-20A, Japan), an automatic sampler (Sil-20A, Japan), and a CTO-20A column temperature chamber, were used for chromatographic analysis. Ethanol extracts of Radix *S. miltiorrhizae*, TIIA, TI, CT, and DH-TI were separated on WondaSil C18–WR (250 mm × 4.6 mm, 5 μm) at 30 °C. The sample was eluted at a flow rate of 1 mL/min in a gradient elution program of A (acetonitrile) and B (water): 0–0.01 min (42% B); 0.01–25 min (10% B); 25–27 min (42% B); 27–35 min (42% B). The injection volume was 20 μL. Monitoring was performed at 270 nm with an SPD-M20A diode array detector. TIIA, TI, CT, and DH-TI.

### Cell lines and culture

Human HCC cell lines MHCC97-H and HepG2 were obtained from the Chinese Academy of Sciences cell bank, Beijing, China. Cells were cultured in DMEM/high glucose medium with 10% FBS and 100 U/mL penicillin–streptomycin, and incubated at 37 °C in a 5% CO_2_ incubator.

### Cell viability assay

Cells were seeded into a 96-well plate at a density of 1 × 10^5^/mL (100 μL/well) and cultured overnight. Cell viability was evaluated by MTT (5 mg/mL) at 24 h. Following the addition of 10 μL MTT per well, cells were incubated for 4 h at 37 °C. The medium was removed, and 150 μL of dimethyl sulfoxide (DMSO) was added to each well. The optical density (OD) value was detected at the wavelength of 490 nm using a microplate reader (Thermo Fisher, USA) after thoroughly shaking the plate.

### Colony formation assay

One-thousand cells were seeded in a six-well plate and cultured in DMEM containing 10% FBS. The culture medium was replaced every 3 days. After 14 days, the colonies were fixed with 4% paraformaldehyde for 10–15 min and stained with crystal violet solution for 10 min. Cell colonies in each well that had ≥ 50 cells per colony were counted after washing with water.

### Wound-healing assay

MHCC97-H and HepG2 cells (4 × 10^5^ cells/well) were seeded in a 6-well plate for use in wound-healing assays. After the cells reached full confluence, the cell monolayer was scratched using a 10 μL pipette tip and the medium was replaced with medium containing 0.1% FBS. The scratches were imaged at 0 and 48 h following scratching, using a 10× objective microscope. The cell migration rate was calculated as the distance of the wound recovered versus that of the original wound.

### Transwell migration and invasion assay

A Transwell invasion and migration assay was performed to determine cell migration and invasion capacities. Cells (2 × 10^5^) in serum-free medium were seeded onto the upper chamber after drug treatment, and the bottom chamber was placed into DMEM medium containing 10% FBS. After 24 h incubation, cells on the upper surface of the membrane were removed and the invading cells were fixed in 4% paraformaldehyde for 10 min and stained with crystal violet solution for 5 min. A cell invasion assay was performed similarly, except that 60 μL of Matrigel (BD Biosciences, USA) diluted 1:8 with serum-free medium, was added to each well overnight before the cells were seeded onto the membrane. The number of invading cells was analyzed statistically using three independent experiments and the results were averaged from five image fields.

### Flow cytometry for cell cycle analysis

MHCC97-H and HepG2 cells were harvested by trypsinization following treatment with RSM (0, 5, 10, and 20 μg/ml) for 24 h. Cells were fixed in 70% cold ethanol at 4 °C overnight after washing twice in cold PBS. Then, the cells were washed twice with cold PBS, centrifuged at 1000*g* for 3–5 min to precipitate cells and then treated with 500 μL staining buffer (PI/RNase A = 9:1) at 37 °C for 40 min in the dark. A flow cytometer was used to analyze the samples and the results were analyzed using Modifit acquisition software.

### Flow cytometry for analysis of cell apoptosis

Apoptosis was analyzed using an Annexin V-FITC apoptosis detection kit (Vazyme, China). MHCC97-H and HepG2 cells were seeded at a density of 10^6^ cells/well in 6-well plates. After 24 h treatment with RSM, the cells were analyzed using an Annexin V-FITC kit to detect apoptosis. The apoptotic cells were detected by flow cytometry (BD Biosciences, USA).

### Western blot

Cell proteins were extracted after 24 h incubation with the indicated concentrations of RSM. The cells were homogenized and lysed in sample buffer (0.5 M Tris/HCl pH 6.8, 50% glycerol, 10% sodium dodecyl sulphate [SDS], 1: 100 protease and phosphatase inhibitor cocktail). Total cell lysates were separated by 10% sodium dodecyl sulfate polyacrylamide gel electrophoresis (SDS-PAGE) and t transferred onto polyvinylidene difluoride (PVDF) membranes (Bio-Rad, USA). Then, the membranes were blocked with 5% bovine serum albumin for 2 h at room temperature and incubated with the appropriate primary antibody solution including EFGR (1:1000), anti-PI3-kinase p85-α (1:1000), anti-Bcl-2 (1:1000), anti-Bax (1:1000), anti-AKT (1:1000), anti-p-AKT (1:1000), and β-actin (1:8000) overnight at 4 °C. The membranes were incubated with HRP-conjugated goat anti-mouse or anti-rabbit IgG (1:1000) for 2 h at room temperature and the protein bands were visualized by an ECL kit.

### Orthotopic transplantation of HCC in nude mice

Five-week-old BALB/c male nude mice were obtained from the Experimental Animal Center of Guangzhou University of Traditional Chinese Medicine. MHCC-97L-luciferase cells (5 × 10^6^/100 μL) were subcutaneously injected into the nude mice. Once it reached a diameter of 1 cm, the subcutaneous tumor was cut into approximately 1 mm^3^ pieces and implanted into the left liver lobes of another group of nude mice. Mice with luciferase signals from liver HCC tumors were analyzed by Xenogen IVIS after 7 days. Surviving mice, with successfully implanted tumors confirmed by in vivo imaging (Xenogen Corp. USA), were randomly divided into three groups: a control saline group (n = 5), an RSM low dose group (n = 5, 100 mg/kg/day), and an RSM high dose group (n = 5, 200 mg/kg/day). Nude mice were anesthetized with sodium pentobarbital (50 mg/kg i.p.) at the end of the study. To quantify tumor progression, in vivo imaging of liver tumors was performed by Xenogen IVIS 2, 3, and 4 weeks after tumor implantation. All animal protocols in the study were performed in accordance with international ethical guidelines and the National Institutes of Health Guide concerning the Care and Use of Laboratory Animals. All experiments and the use of animals in this study was approved by the Institutional Animal Care and Use Committee of Guagnzhou University of Traditional Chinese medicine, China.

### Statistical analysis

All statistical analyzes were performed by Statistical Product and Service Solutions (SPSS) 20.0 software. One-way analysis of variance (ANOVA) followed by the Dunnett’s post hoc test was used for multiple groups comparisons. Our data are expressed as mean ± standard deviation (SD). A value of *P* < 0.05 was considered significant.

## Results

### HPLC analysis of RSM

**Fig. 2:**
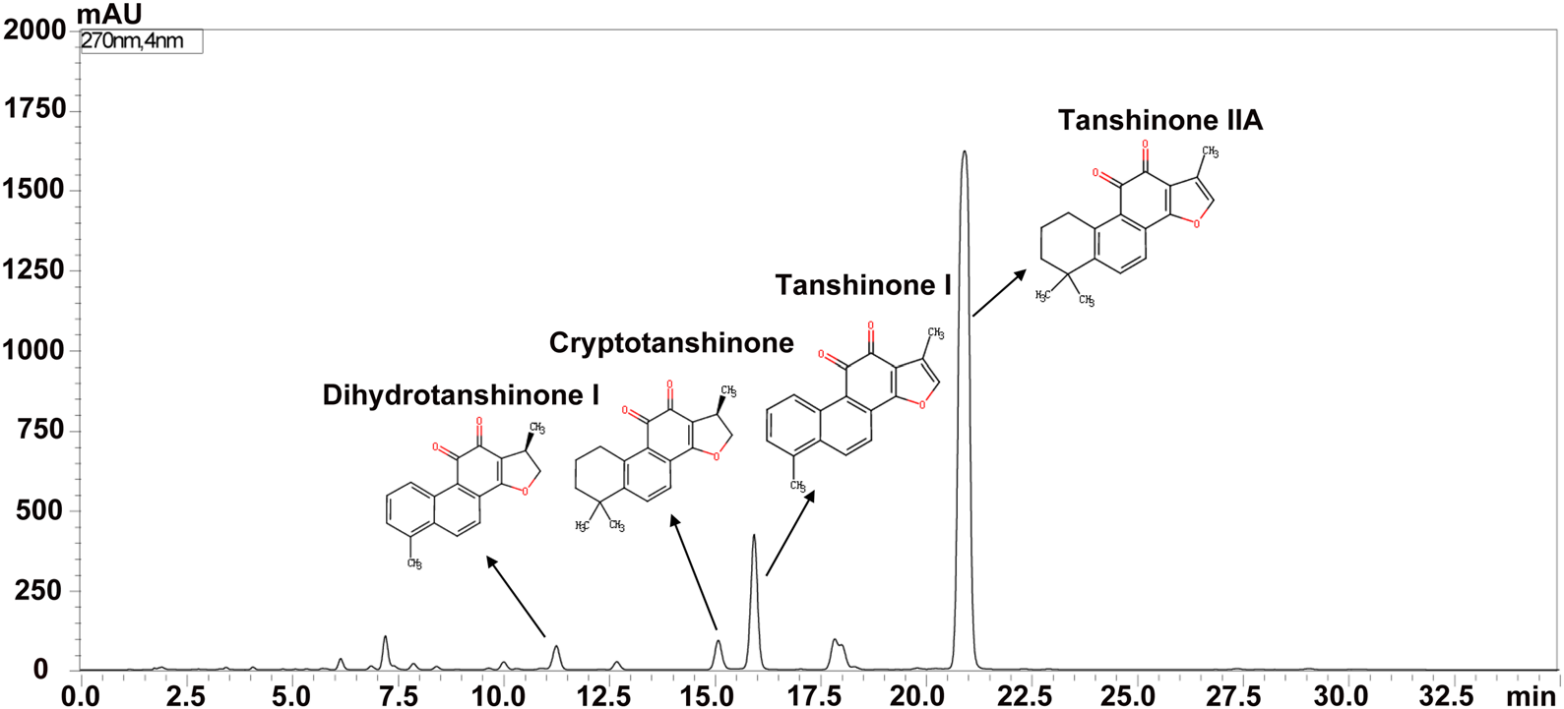
RSM analyzed by HPLC. From right to left, the four major peaks represent: tanshinone IIA, tanshinone I, cryptotanshinone, and dihydrotanshinone I

The constituents of the ethanol extracts of RSM were analyzed and identified by HPLC. As shown in Fig. 2, compounds represented by four main peaks of the HPLC chromatogram were identified as TIIA, TI, CT, and DH-TI.

### Chemical composition and targets of RSM

In total, 202 compounds were collected from RSM. Sixty-five candidate molecules were obtained with OB ≥ 30% and DL ≥ 0.18. Notably, the OB values of salvianolic acid B and salvianolic acid A were lower than 30%; however, both are widely used to treat liver disease in vivo [32, 33] and in vitro [34, 35]. Therefore, these two molecules were also employed as candidate molecules, making a total of 67 active ingredients. Detailed information on active molecules in RSM is presented in Additional file 1: Table S1. Among the 67 compounds obtained, five candidate molecules had no targets. Finally, 62 chemical compounds yielded 101 putative targets. The details of these targets are shown in Additional file 2: Table S2.

### Compound-compound targets network analysis

**Fig. 3:**
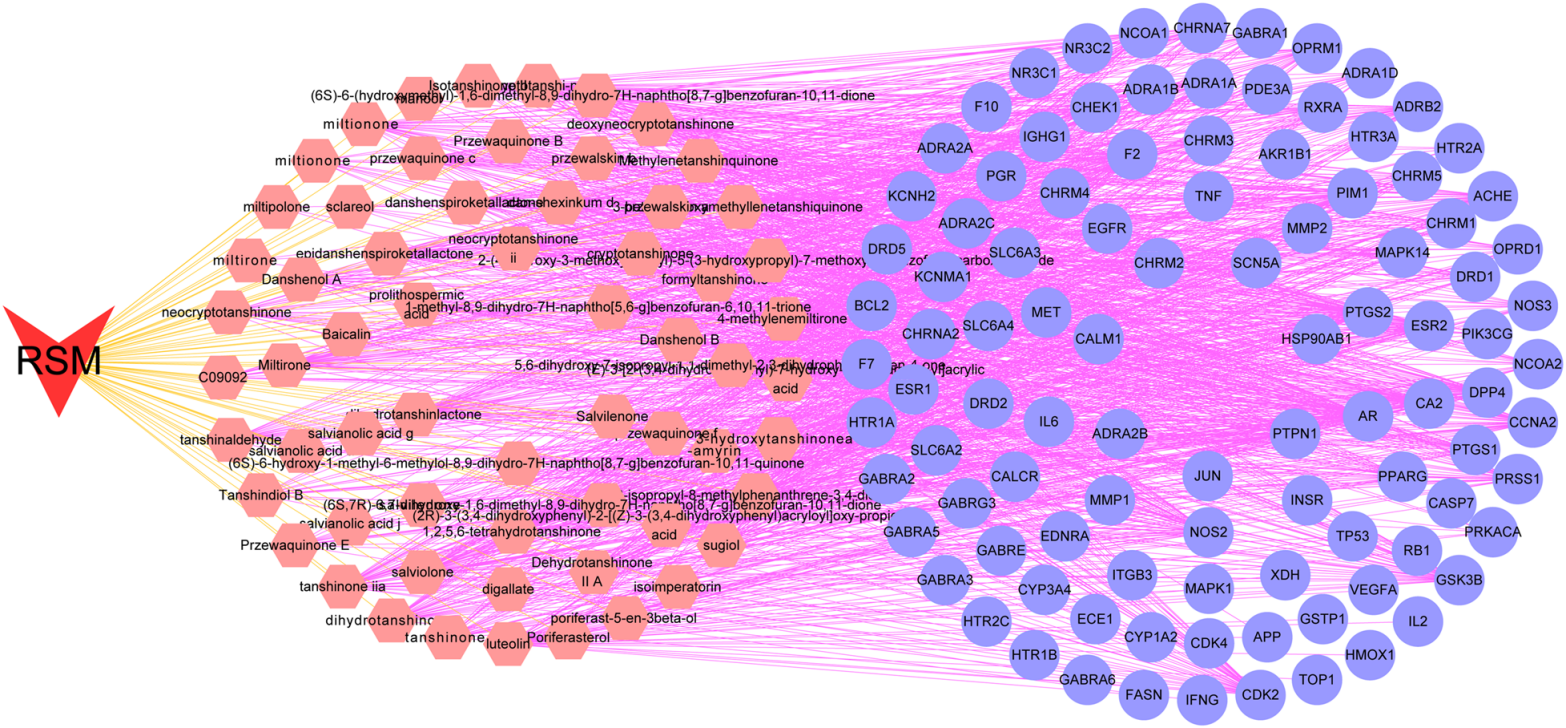
Compound-compound target network. RSM consists of 62 compounds and 101 compound targets (red V represents RSM; pink hexagons represent compounds of RSM; blue circles represent compound targets)

RSM exerts a wide range of biological and pharmacological effects through a variety of molecules and targets. To elucidate the complex interactions of RSM constituents and their corresponding targets at the system level, we constructed a network based on the potential compounds of RSM and candidate targets. The network consists of 163 nodes (62 compound nodes and 101 compound target nodes) and 1322 edges (Fig. 3). In Fig. 3, red V represents RSM, pink hexagons represent compounds of RSM, and blue circles represent compound targets. The average value (the number of connections that each node has to other nodes) of the candidate compounds was 21.3, and the average value of the 19 compounds was greater than 30, indicating that RSM modulates multiple targets by multiple components to exert various therapeutic effects. These are potential active compounds of RSM due to their key positions in this network: tanshinone IIA (degree = 40) and cryptotanshinone (degree = 35). Taking tanshinone IIA as an example, this molecule exhibits extensive pharmacological activities, with anti-coagulation, anti-apoptosis, and anti-oxidative properties, by mediating the activation of retinoid X receptor alpha 1, inducible nitric oxide synthase, and BCL2, the apoptosis regulator [36]. Additionally, the results suggested that many targets were hit by multiple compounds in the network. ESR1, AR, and PTGS2 are targeted by 58, 55, and 50 compounds. These targets play critical roles in processes such as cell proliferation and inflammation [37, 38].

### Identification of candidate protein targets associated with HCC

Overall, 5730 HCC-related targets were obtained from the GeneCards database, and 153 HCC-related targets were collected from the OMIM database. In total, 5617 HCC-related targets were identified following the deletion of duplicate targets. To elucidate the pharmacological mechanism of action of RSM in human HCC, a PPI network was determined and GO and pathway enrichment analysis was performed on the common targets of RSM and HCC. Seventy-two common targets were obtained. The details of targets are shown in Additional file 3: Table S3.

### PPI network

**Fig. 4:**
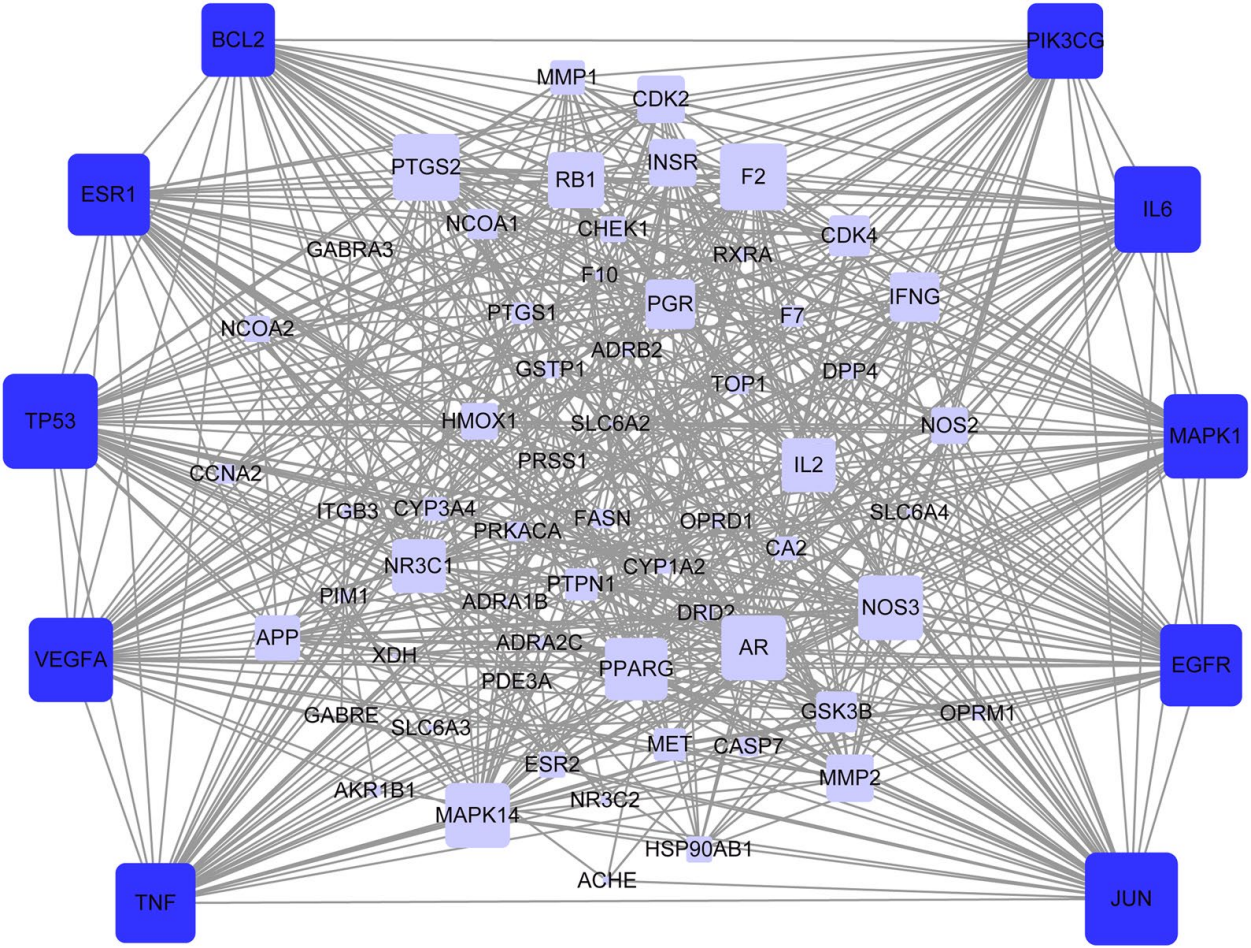
Protein-protein interaction (PPI) network. The nodes get larger with increasing degree. Edges: PPIs between putative targets of RSM and their interactive partners; blue round rectangular nodes: common targets of RSM and HCC; dark blue round rectangular nodes: hub targets of RSM and HCC

We constructed a protein-protein interaction network, which denoted the relationship among 72 HCC-related targets to determine the importance of candidate targets. One target was no gene-gene interaction, and another target was not found in the above two databases. The network contained 70 nodes and 571 edges (Fig. 4). A hub node was defined as two-times greater than the average node degree in the network [39]. The average node degree (the number of targets associated with it) of the HCC-related targets was 32.6. Therefore, the hub nodes including TNF, PIK3CG, VEGFA, IL6, EGFR, MAPK1, JUN, TP53, BCL2, and ESR1 contained over 32.6 degrees. The number of edges per node as somewhat large (44 in TP53, 43 in JUN, 40 in IL6, 39 in MAPK1, 39 in VEGFA, 38 in ESR1, 38 in EGFR, 37 in TNF, 35 in PIK3CG, and 34 in BCL2). The network results suggest that the abovementioned genes may represent hub genes for the development of HCC. EGFR is expressed at high levels in cancer cells, and its activation EGFR appears to be important for tumor progression and growth [40]. Thus, RSM may act on HCC by targeting EGFR.

### Gene ontology and pathway enrichment analysis for targets

**Fig. 5:**
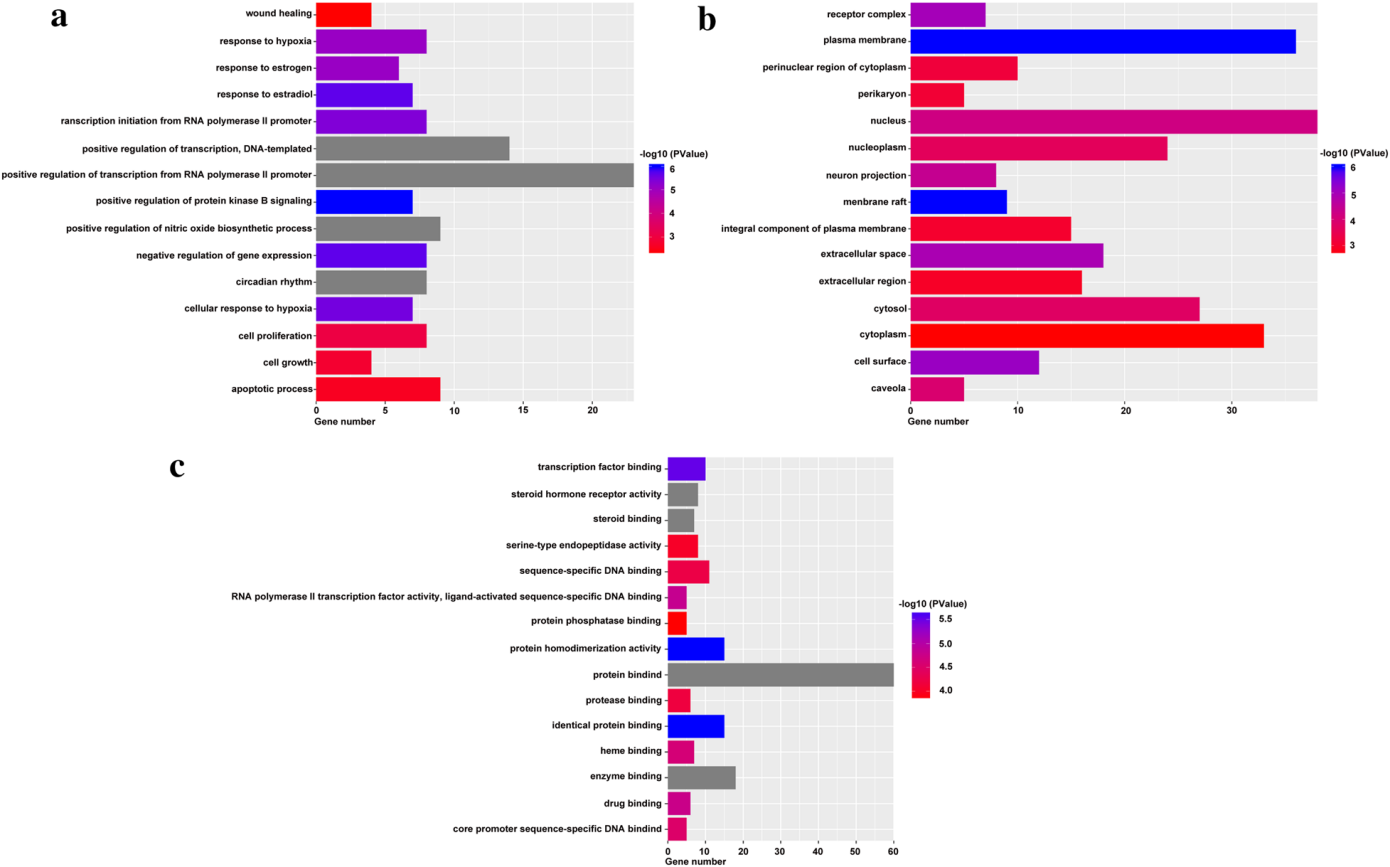
Gene ontology enrichment analysis of identified related targets. **a** Biological process, **b** cellular component, **c** molecular function

**Fig. 6:**
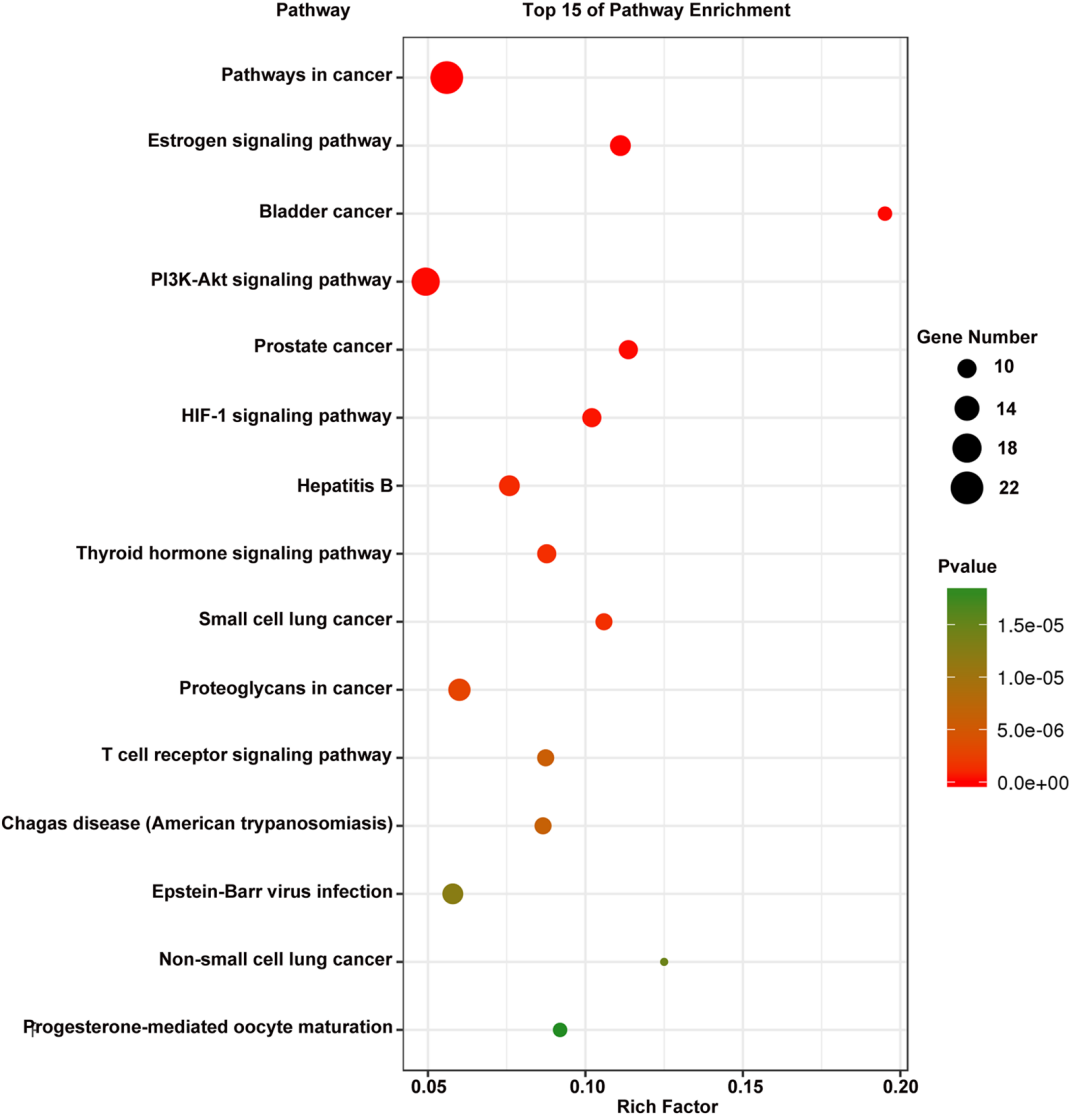
Pathway analysis for identified related targets

To elucidate the biological characteristics of putative targets, functional enrichment analysis was performed for the target genes. The results, and detailed information on the GO targets, and pathways are shown in Additional file 4: Table S4. The 15 most significantly enriched terms in biological process (BP), molecular function (MF), and cellular component (CC) categories (*P* < 0.05) are listed in Fig. 5. Figure 5 indicates that RSM may regulate cell proliferation, cell growth, and apoptotic processes, via protein binding, enzyme binding, and protease binding in the plasma membrane, nucleus, and cytoplasm to exert anti-HCC potential. A pathway analysis was performed to explore the underlying mechanisms of RSM action for treatment of HCC. The results demonstrated that 68 targets were mapped into the 99 KEGG pathways, including pathways in cancer, PI3K/AKT signaling, and proteoglycans in cancer, while four of 72 targets were not mapped into pathways. The 15 most significant pathways (*P* < 0.05) are shown in Fig. 6. Cancer signaling pathways displayed the highest number of target connections (degree = 22), followed by PI3K/AKT signaling pathways with 17 targets, and proteoglycans in cancer with 12 targets. The PI3K/AKT pathway is closely related to the proliferation, differentiation, apoptosis, migration, and adhesion of tumor cells involving multiple targets, including EGFR, BCL2, GSK3B, CDK2, and CDK4. Therefore, the data provided theoretical evidence that RSM may activate P13K/AKT signaling pathways in HCC.

### RSM inhibits the proliferation of human HCC cells

**Fig. 7:**
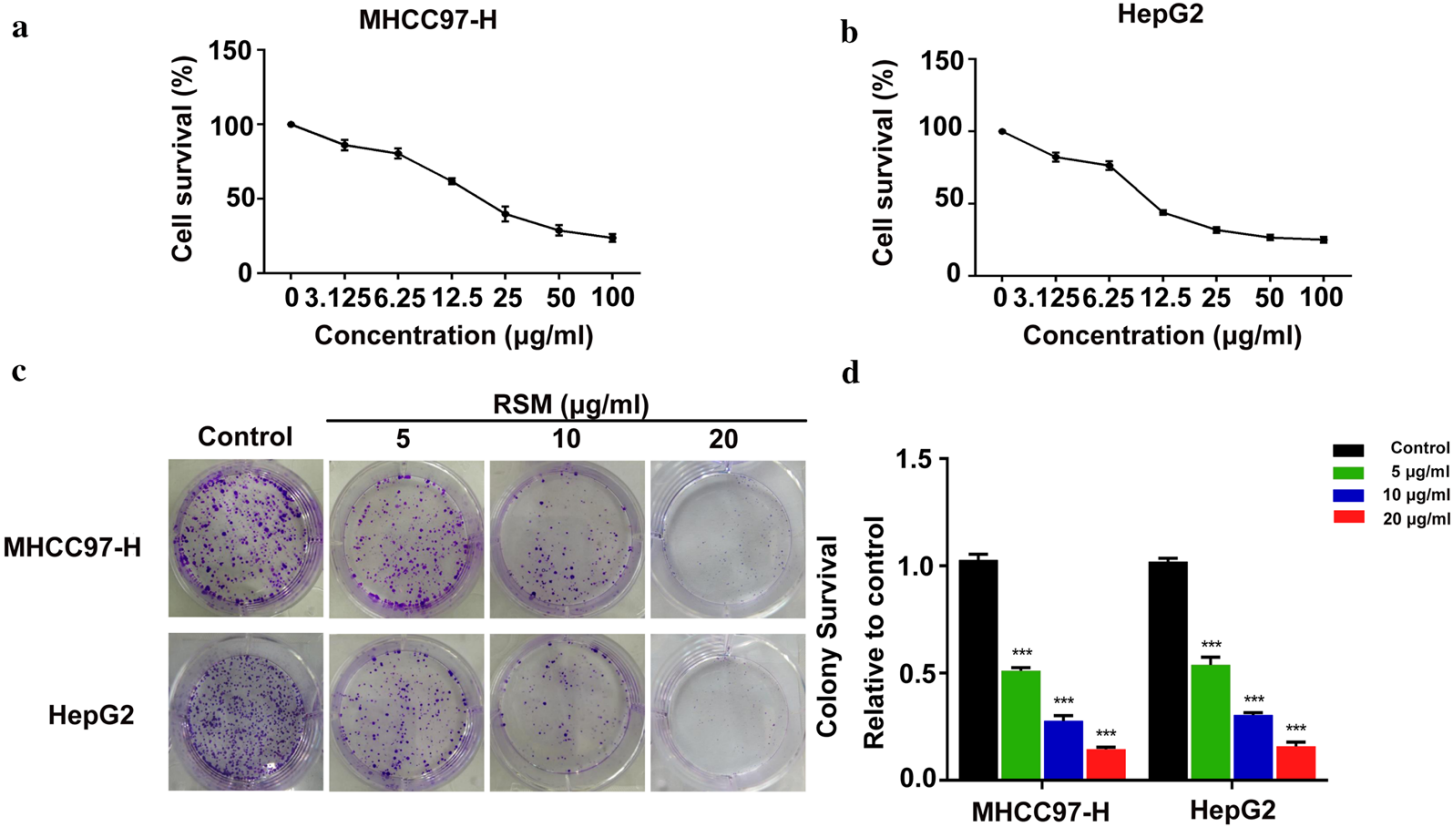
RSM inhibits cell viability. **a** Viability of MHCC97-H and **b** HepG2 cells following 24 h incubation with the indicated doses of RSM. All data are presented as mean ± standard deviation (SD; n = 4). **c** Colony formation assay was performed using MHCC97-H and HepG2 cells treated with RSM. A representative of three experiments is shown. **d** Colony formation is presented as mean ± SD (n = 3), *P < 0.05, **P < 0.01, ***P < 0.001 versus the control group

To explore the effects of RSM on human HCC cells, MHCC97-H and HepG2 cells were incubated with increasing doses of RSM for 24 h. Cell viability was assessed by MTT assay. The results of the MTT assay suggested that RSM significantly inhibited the viability of MHCC97-H and HepG2 cells (Fig. 7a, b). In a colony formation assay, RSM decreased colony formation at all indicated doses (Fig. 7c, d). These data suggested that RSM can inhibit HCC cell viability and proliferation.

### RSM induces cellular apoptosis and arrests cell cycle progression in the G2/M phase

**Fig. 8:**
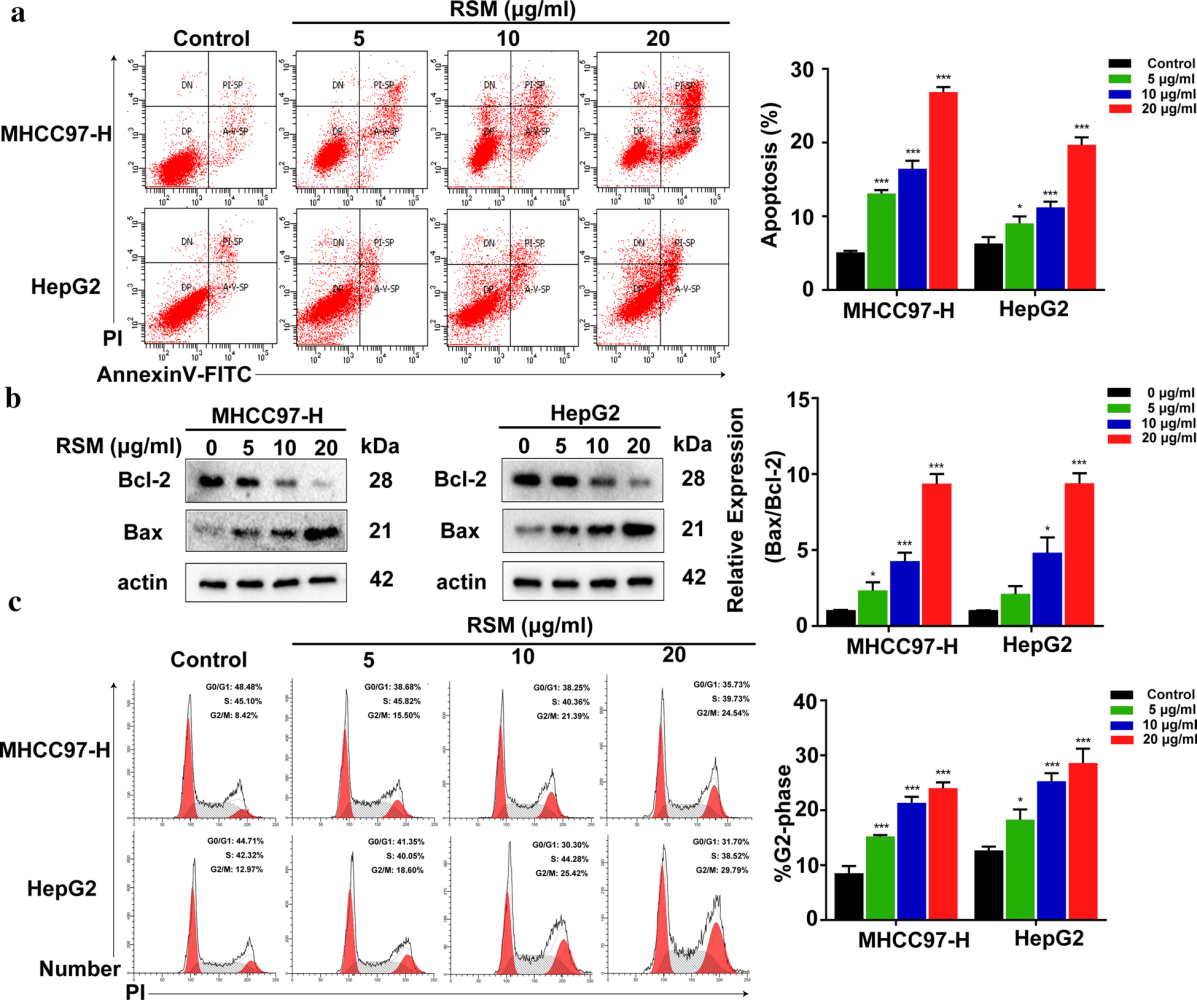
RSM induces apoptosis and cell cycle G2/M arrest in MHCC97-H and HepG2 cells. **a** Flow cytometric analysis of Annexin V/PI double staining in MHCC97-H and HepG2 cells treated with various concentrations of RSM for 24 h. **b** MHCC97-H and HepG2 cells were treated with RSM at the indicated concentrations for 24 h and subjected to western blotting with antibodies against Bcl-2 and Bax. **c** Cell cycle analysis revealed that RSM arrested cells at the G2/M checkpoint. MHCC97-H and HepG2 cells were incubated with the indicated dose of RSM for 24 h, stained with Propidium iodide (PI), and DNA content was analyzed via flow cytometry. All data are presented as mean ± SD (n = 3), *P < 0.05, **P < 0.01, ***P < 0.001 versus the control group

We next investigated whether RSM affects cell cycle arrest and cellular apoptosis via flow cytometry. Administration of RSM at 5–20 μg/mL led to a significant increase in the number of apoptotic cells (Fig. 8a). As shown in Fig. 8b, we also assessed RSM-induced apoptosis by western blot. The results suggested that the expression of the apoptosis-related protein Bcl-2 decreased and Bax increased after treatment with RSM. Furthermore, RSM induced cell cycle arrest in MHCC97-H and HepG2 cells (Fig. 8c). These results suggest that RSM might induce apoptosis and cell cycle arrest.

### RSM inhibits human HCC cell migration and invasion

**Fig. 9:**
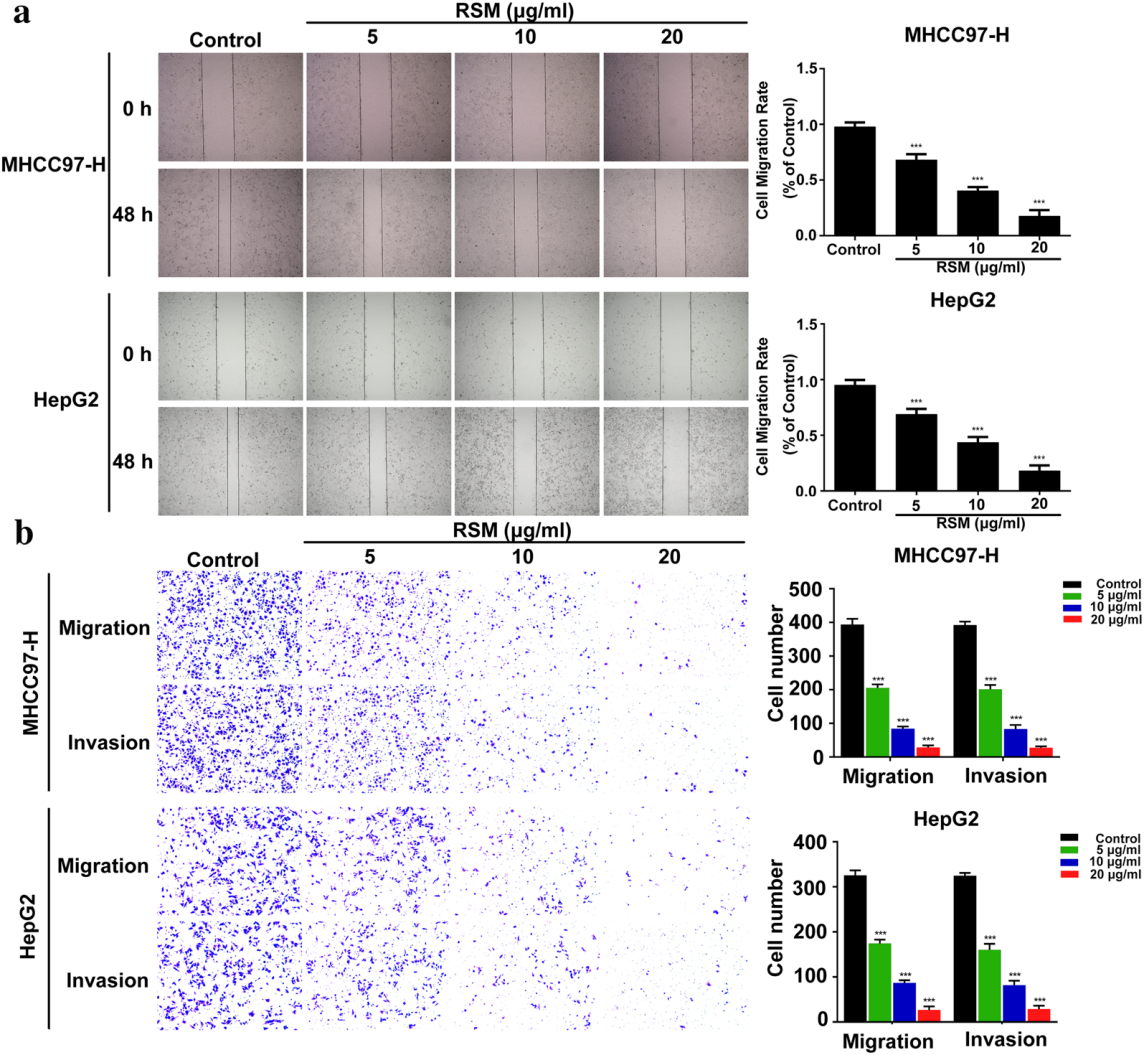
RSM inhibits the migration and invasion of MHCC97-H and HepG2 cells. **a** Effects of RSM on the migration of MHCC97-H and HepG2 cells as shown by a wound healing assay. The cell surface was wounded and cells were photographed at 0 and 48 h (×100 magnification). **b** Transwell migration and invasion assays were performed to determine the effect of RSM on cell migration and invasion. Invading and migrating cells were photographed (×100 magnification). All data are presented as mean ± SD (n = 3), *P < 0.05, **P < 0.01, ***P < 0.001 versus the control group

To investigate the migratory ability of RSM in human HCC cells, wound healing and Transwell migration assays were performed. As shown in Fig. 9a, unlike in the control group, migration was markedly inhibited in cells treated with RSM. The Transwell migration assay also showed that RSM treatment inhibited the migration of cells in a dose–dependent manner (Fig. 9b). Moreover, the treatment of MHCC97-H and HepG2 cells with 5–20 μg/mL RSM for 24 h inhibited the number of invading cells (Fig. 9b). These results confirmed that RSM inhibited the migration and invasion of cancer cells.

### RSM down-regulates EGFR expression, and attenuates PI3K/AKT signaling in human HCC cells

**Fig. 10:**
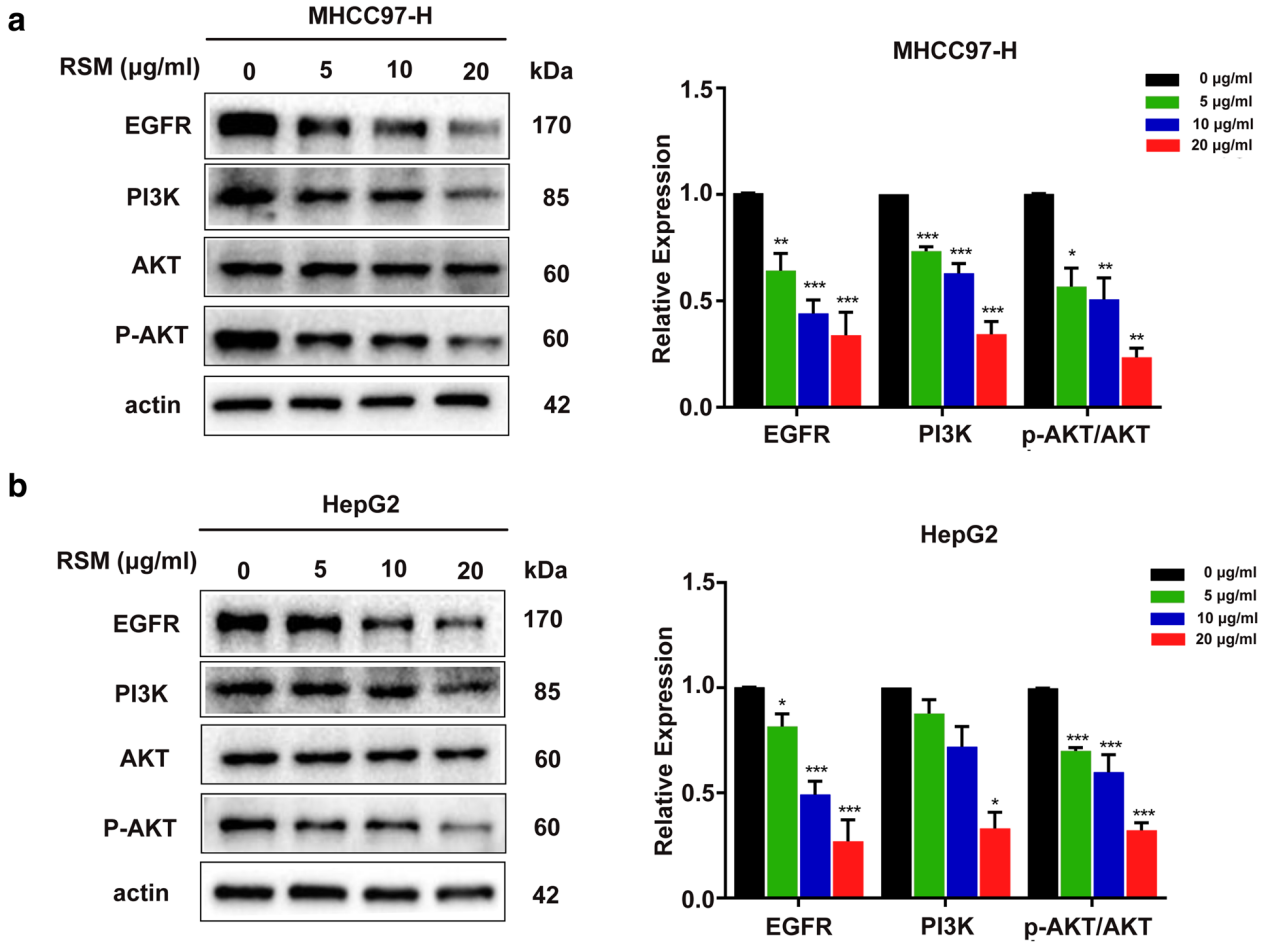
RSM down-regulates EGFR expression, and attenuates PI3K/AKT signaling in human HCC cells. Western blot analysis of EGFR, PI3K, AKT, and p-AKT in **a** MHCC97-H and **b** HepG2 cells. Data are presented as mean ± SD (n = 3), *P < 0.05, **P < 0.01, ***P < 0.001 versus the control group

The network pharmacology results described above, suggested that the EGFR and PI3K/AKT signaling pathways might account for the mechanism of RSM action in on HCC. Therefore, we evaluated the expression levels of EGFR, PI3K, AKT, and p-AKT by western blot. As shown in Fig. 10, the expression of EGFR decreased in both MHCC97-H and HepG2 cells treated with 5–20 μg/mL RSM for 24 h. Next, we assessed the protein levels of PI3K, AKT, and p-AKT. The results indicated that RSM significantly decreased the expression of p-AKT and PI3K, whilst total AKT expression was not changed. Taken together, these results suggest that the effect of RSM in HCC might be related to the EGFR and PI3K/AKT signal transduction pathways in human HCC cells.

### RSM inhibits the growth of liver tumors in nude mice following orthotopic liver transplantation

**Fig. 11:**
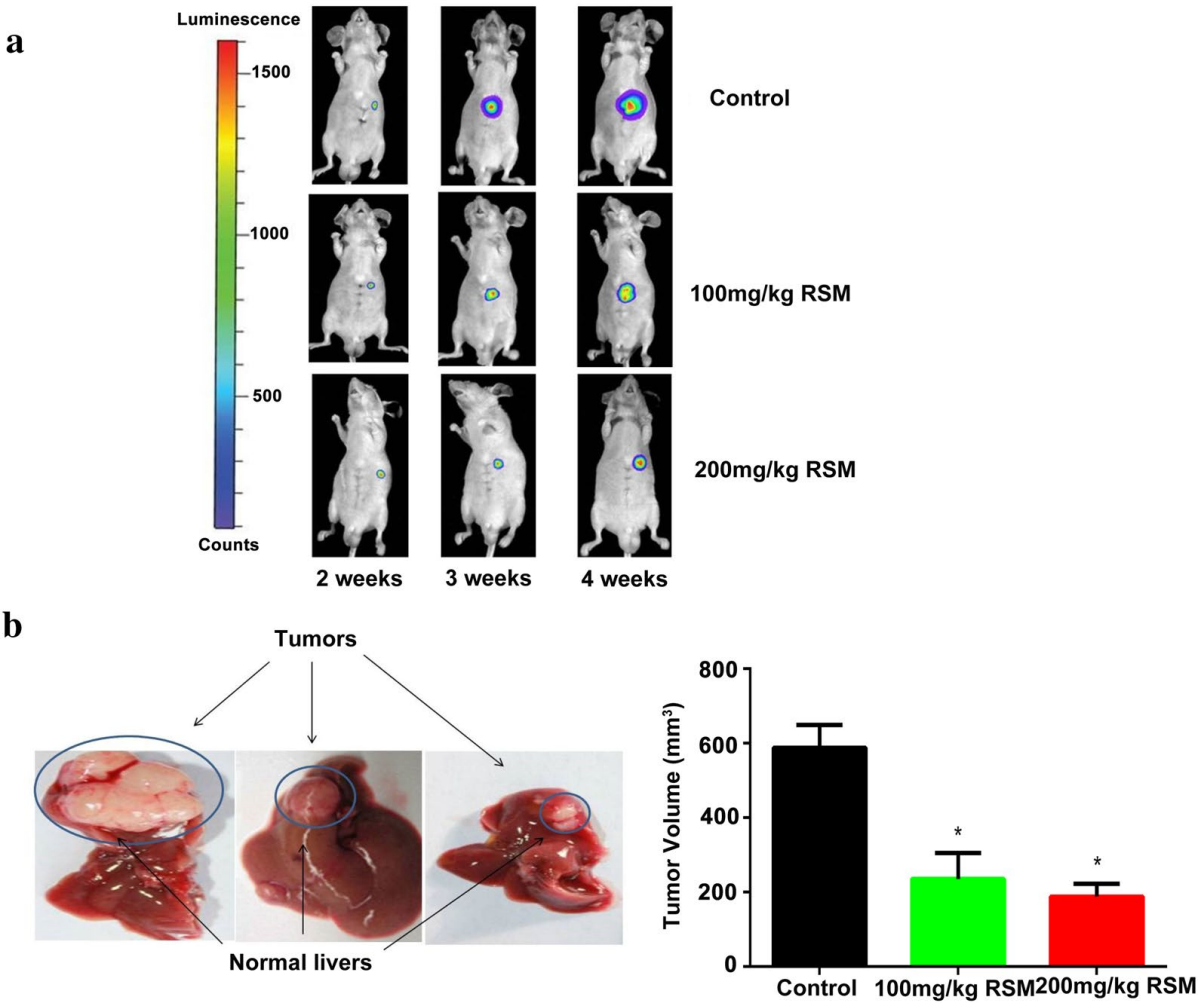
RSM suppresses the growth of liver tumors in nude mice orthotopically implanted with HCC. **a** In vivo detection of liver luciferase signals revealed a significant inhibition of luminescence in RSM-treated mice compared with control mice 3 weeks after implantation. **b** There was a significant change in the volume of orthotopic transplanted liver tumors following RSM treatment (*P* < 0.05). Data are presented as mean ± SD (n = 5), *P < 0.05 versus the control group

We explored the anti-HCC potential of RSM in vivo. Implantation of MHCC-97L-luciferase facilitated monitoring of liver cancer progression by bioluminescence imaging to quantify bioluminescence emitted from cancer cells. Three weeks after implantation, there was a significant inhibition in luminescence in the livers of mice in the RSM (100 mg/kg, 200 mg/kg) group compared to that in the control group, as visualized using Xenogen imaging (Fig. 11a). The nude mice were sacrificed after 4 weeks and the size of orthotopic liver transplantations were measured in each group. The tumor volume of the orthotopic liver transplantation decreased significantly after RSM treatment (*P* < 0.05) (Fig. 11b).

## Discussion

In this study, RSM inhibited cellular proliferation, invasion, and migration, and induced cell cycle arrest at the G2/M phase and cellular apoptosis associated with HCC in vitro through modulation of the PI3K/AKT pathway and EGFR. RSM is one of the most popular Chinese medicinal herbs. Studies have demonstrated that RSM or RSM extracts may have positive effects in patients with liver fibrosis [41, 42], dementia [43–45], osteoporosis [46], chronic renal failure [47–49], and fulminant hepatic failure [50]. Previous research has shown that RSM extracts inhibit proliferation and induce apoptosis in HepG2 cells [51]. However, the underlying mechanisms of the pharmacological action of RSM in HCC therapy remain unclear.

We analyzed the putative active compounds and targets of RSM through a network pharmacology approach, a powerful tool that has emerged to better understand the underlying mechanisms of action of TCM. To elucidate the pharmacological mechanism of RSM acting on human HCC, we performed a PPI network analysis. The PPI network analysis revealed that TNF, PIK3CG, VEGFA, IL6, EGFR, MAPK1, JUN, TP53, BCL2, and ESR1 may represent hub genes against HCC. EGFR is a transmembrane receptor tyrosine kinase and a receptor for members of the ErbB family of extracellular protein ligands [52]. EGFR signaling modulates diverse cell functions and promotes cellular proliferation, differentiation, migration, growth, and survival [53]. Mutations in EFGR lead to its overexpression (upregulation or amplification), which is associated with many cancers, including lung cell carcinoma [54], glioblastoma [55], anal cancer [56], and epithelial tumors in the head and neck [57]. Previous studies have indicated that EGFR activation plays a pivotal role in the development of HCC [58, 59]; however, there is no evidence to support the downregulation of EGFR expression by RSM. In this study, we evaluated EGFR expression in MHCC97-H and HepG2 cells. The results showed that RSM could decrease EGFR expression in a dose-dependent manner compared to that in the control group. Furthermore, EGFR is also an upstream protein in the PI3K/AKT signal transduction pathway, which is an important target in cancer research [60, 61].

The pathway analysis results obtained by DAVID suggested that pathways in cancer, PI3K/AKT signaling pathway, and proteoglycans in cancer may be closely related to HCC progression. Phosphatidylinositol-3-kinases (PI3Ks) are a family of enzymes that phosphorylate the 3-position hydroxyl group of the inositol ring of phosphatidylinositol. PI3K contains two domains: a catalytic domain, P110, and a regulatory domain, P85 [62]. AKT, also referred to as protein kinase B, is a serine/threonine-specific protein kinase that plays a critical role in multiple cellular processes. The PI3K/AKT signaling pathway plays a key role in normal cellular processes involved in growth, glucose metabolism, proliferation, survival, apoptosis, motility, ribosomal function, gene transcription, migration, and invasion, via phosphorylation of a variety of substrates by the serine/threonine kinase AKT [63, 64]. A growing body of evidence suggests that aberrant activation of the PI3K/AKT pathway promotes the development and progression of multiple human malignancies [65, 66], such as lung cancer [67], ovarian cancer [68], and oral squamous cell carcinoma [69]. Cryptotanshinone, a natural active compound of *S. miltiorrhiza* Bunge, has been shown to affect cell cycle arrest and apoptosis through the PI3K/Akt/NFκB and JAK2/STAT3 pathways in cholangiocarcinoma cells [70]. Tanshinone IIA, one of the main compounds of *S. miltiorrhiza* Bunge, has been shown to inhibit the growth of cancer cells via autophagy and apoptosis, which is related to the Beclin-1/Atg7/Atg12-Atg5 and PI3K/Akt/mTOR pathways [71]. In the present study, high expression of phosphorylate AKT (p-AKT) and PI3K in the control group indicated the activation of the PI3K/AKT signal transduction pathway. Compared to levels in the control group, the levels of p-AKT and PI3K in the treatment group were significantly decreased, and the expression of total AKT remained constant. Therefore, our results revealed that RSM plays a significant anti-HCC role, which is mediated by the PI3K/AKT pathway.

This study demonstrates, for the first time, that RSM can efficiently suppress cellular proliferation and induce G2/M checkpoint arrest and apoptosis. In addition, RSM also affects the migration and invasion ability of MHCC97-H and HepG2 cells. Taken together, our results indicate that RSM acts on HCC through the EGFR and PI3K/AKT signaling pathways. Due to budgetary and time constraints, only a few key molecules and mechanisms involved in the antitumor activity of RSM on HCC were studied here. Therefore, other potential mechanisms that may underly the antitumor action of RSM in vitro and in vivo await further elucidation. Despite its limitations, this study provides powerful and preliminary data to support the future evaluation of RSM in HCC. RSM may be a potential anti-HCC inhibitor, which can be developed as a therapeutic option for the treatment of cancer.

## Conclusions

Taken together, our studies illustrate that RSM could suppress proliferation, induce G2/M checkpoint arrest and apoptosis, and impair the migration and invasion ability of MHCC97-H and HepG2 cells in vitro, which may be linked to the EGFR and PI3K/AKT signaling pathways. In addition, RSM could inhibit liver tumor growth of orthotopically implanted HCC in nude mice.

## Additional files

**Additional file 1: Table S1.** List of active ingredients in Radix *Salviae Miltiorrhizae* (RSM).

**Additional file 2: Table S2.** List of the chemical compounds and putative targets of RSM following screening.

**Additional file 3: Table S3.** List of hepatocellular carcinoma (HCC)-related targets identified from the GeneCards and OMIM databases and common targets of RSM and HCC.

**Additional file 4: Table S4.** Gene ontology (GO) terms and pathways.

## Data Availability

The datasets used and/or analyzed during the current study are available from the corresponding author on reasonable request.

## Abbreviations

RSM: Radix *Salviae Miltiorrhizae*
HCC: human hepatocellular carcinoma
EGFR: epidermal growth factor receptor
PI3K: phosphatidylinositol 3-kinase
AKT: protein kinase B
Bcl-2: B-cell lymphoma-2
TIIA: tanshinone IIA
CT: cryptotanshinone
DH-TI: dihydrotanshinone I
TI: tanshinone I
VEGFR: vascular endothelial growth factor receptor
HER2: human epidermal growth factor receptor-2
MEK: mitogen-activated protein kinase kinase
ERK: extracellular signal-regulated kinase
PARP: poly ADP-ribose polymerase
mTOR: mammalian target of rapamycin
TCM: traditional chinese medicine
TCMSP: Traditional Chinese Medicine Systems Pharmacology
ADME: absorption, distribution, metabolism, and excretion
DL: drug-likeness
OB: oral bioavailability
OMIM: Online Mendelian Inheritance in Man
DMEM: Dulbecco’s modified Eagle’s medium
FBS: fetal bovine serum
HPLC: high-performance liquid chromatography
PBS: phosphate-buffered saline
PI: propidium iodide
FITC: fluorescein
SDS-PAGE: sodium dodecyl sulfate polyacrylamide gel electrophoresis
PVDF: polyvinylidene difluoride
BP: biological process
MF: molecular function
CC: cellular component
DAVID: The Database for Annotation, Visualization and Integrated Discovery
